# Velo-pharyngeal dysfunction: Evaluation and management

**DOI:** 10.4103/0970-0358.57201

**Published:** 2009-10

**Authors:** Jeffrey L. Marsh

**Affiliations:** Department of Plastic Surgery, St. Louis University School of Medicine, St. Louis MO, USA

**Keywords:** Velo-pharyngeal dysfunction, Velo-pharyngeal insufficiency, Velo-pharyngeal incompetency

## Abstract

Separation of the nasal and oral cavities by dynamic closure of the velo-pharyngeal port is necessary for normal speech and swallowing. Velo-pharyngeal dysfunction (VPD) may either follow repair of a cleft palate or be independent of clefting. While the diagnosis of VPD is made by audiologic perceptual evaluation of speech, identification of the mechanism of the dysfunction requires instrumental visualization of the velo-pharyngeal port during specific speech tasks. Matching the specific intervention for management of VPD with the type of dysfunction, i.e. differential management for differential diagnosis, maximizes the result while minimizing the morbidity of the intervention.

## INTRODUCTION

Dynamic separation of the nasal cavity from the oral cavity is a necessary component in the production of normal speech. This separation occurs in the anatomic space between the nasal and oral cavities known as the velo-pharynx. Failure to achieve this separation not only directly impairs speech with inappropriate escape of air and/or sound into the nose and inability to properly produce specific phonemes, but also indirectly by inducing abnormal compensatory articulations as well as abnormal facial movements. This anatomic locus, the velopharynx, becomes a modifying adjective for the several terms used to describe its dysfunction in literature: velo-pharyngeal insufficiency, velo-pharyngeal incompetency, velo-pharyngeal inadequacy and velo-pharyngeal dysfunction. Velo-pharyngeal dysfunction (VPD) is the preferred nomenclature of this author, and others[1–3] due to the ambiguity of the acronym VPI and association of the various “I” nouns with etiologic specificity. The acronym VP is used both for the noun, velopharynx, and the adjective, velopharyngeal. This article discusses the normal function and dysfunction of the velo-pharynx with respect to evaluation as well as management and outcome of the dysfunction. The unifying theme is differential management for differential diagnosis.

## FUNCTIONAL ANATOMY OF VELO-PHARYNX

The velo-pharynx is the *space* that connects the nasal and oral pharynges. This space is delineated by myomucosal structures: anteriorly – the velum (soft palate), posteriorly – the posterior pharyngeal wall, and laterally – the right and left lateral pharyngeal walls. In a child, the VP space may be statically encroached upon by hypertrophic adenoids, on the posterior pharyngeal wall, and/or hypertrophic tonsils, on the lateral pharyngeal walls. While swallowing and during the production of specific speech phonemes, the muscles surrounding the VP space contract, thereby, moving their overlying mucosa three-dimensionally to separate the nasal and oral cavities by closing the space. Because this motion resembles that of digestive tract sphincters, the zone of dynamic action is often referred to as the *velo-pharyngeal sphincter* even though it lacks discrete, circularly enclosing muscle(s). The space that is dynamically opened and closed is referred to as the *velo-pharyngeal port*. Normal function of the velo-pharynx requires not only closure of the port but also proper coordination and speed of closure as well as re-opening appropriate for the specific task. Incomplete closure, lack of coordination between movement and task, and impaired velocity of closure and/or opening alone or in combination produce *velo-pharyngeal dysfunction (VPD)*. Clinical signs and symptoms of VPD can be direct or indirect. Direct manifestations include: rustling sound during speech (nasal turbulence), inappropriate nasal resonance during speech (hypernasality), and nasal regurgitation during swallowing; indirect manifestations include: abnormal facial movements (grimacing), abnormal phoneme production (compensatory or mal-articulations), and voice disorders (harsh voice ≡ hoarseness, low volume).[4] While in common usage the term VPD connotes a failure of velo-pharyngeal closure for normal physiological tasks, a stenotic velo-pharynx also can produce dysfunction. The clinical signs and symptoms of a non- or minimally patent velo-pharynx include nasal airway obstruction affecting breathing as well as secretions, mouth breathing with chronic open-mouth posture, diminished nasal resonance for appropriate phonemes (hypo- or de-nasality), and obstructive sleep apnoea.

## EVALUATION OF VELO-PHARYNGEAL FUNCTION

All of the clinical signs and symptoms of VPD which aid in its diagnosis can be perceived and documented by a trained observer using auditory and visual perceptual assessments.[5,6] Identification of the anatomic mechanism producing VPD, however, requires a thorough assessment of velo-pharyngeal function through instrumentation.[7–9] If it be felt that differential management of differential diagnosis is ideal, identification of specific patterns of VPD would be useful in optimizing intervention outcomes.[10,11]

Instrumental assessment of velo-pharyngeal function is of two basic types: visualization[12,13] and effect on physical parameters[14–16] Visualization may be done with optics (nasendoscopy) or medical imaging (fluoroscopy or dynamic MRI)[17] Change in physical parameters of sound, airflow, air pressure, and transmitted light have all been reported. Each type of instrumental assessment has assets and limitations. Visualization alone can document the anatomic locus of the dysfunction; however, quantification is difficult and the techniques are cumbersome and expensive. Physical parameters are usually recorded quantitatively and for some can be obtained with minimal disturbance of the subject and at low cost.[18] It is impossible to match a specific VPD management with the anatomic dysfunction and to know the functional outcome of that intervention without pre- and post-intervention visualization. For this reason, functional velo-pharyngeal visualization prior to velo-pharyngeal management has become the standard of care.[19] The most commonly clinically used visualizations are nasendoscopy and video fluoroscopy. These examinations should be conducted by individuals skilled not only in instrumentation (endoscopy, radiology) but also in eliciting standard speech samples designed to assess velo-pharyngeal function in both spontaneous and provocative speech. While both the imaging and speech sampling functions can be performed by the same individuals, the majority of velo-pharyngeal centres utilize two professionals to optimize each aspect of the examination: An instrumental visualizer (endoscopist or radiologist) and a speech pathologist.

## DECISION MAKING FOR MANAGEMENT OF VELO-PHARYNGEAL DYSFUNCTION

Just as inter-disciplinary team care has become the standard for cleft lip/palate and other congenital cranio-facial anomalies, inter-disciplinary team care is indicated for evaluation and management of VPD. The task a specific type of healthcare professional performs may vary from team to team, but competence in speech/language assessment, nasendoscopy, medical imaging, and velopharyngeal surgery is needed. In the velo-pharyngeal assessment team on which I participate, tasks are performed as follows:

Identification of possible VPD - patient's local speech/language pathologist; patient's local ENT; cleft/craniofacial team members from any healthcare discipline

Perceptual speech/language assessment (digital audio/video archived) – cleft/craniofacial team speech/language pathologist

Instrumental velo-pharyngeal assessment (digital audio/video archived) – nasendoscopy (cleft/craniofacial team speech/language pathologist plus cleft/craniofacial team ENT); fluoroscopy (cleft/craniofacial team speech/language pathologist plus radiologist)

Velo-pharyngeal inter-disciplinary team conference to determine treatment recommendation(s) - cleft/craniofacial team speech/language pathologist, cleft/craniofacial team ENT, cleft/craniofacial team plastic surgeon.

The velo-pharyngeal inter-disciplinary team conference reviews the patient's history, views the perceptual and instrumental recordings (including prior recordings when extant), discusses interpretations and treatment options and then comes to a consensus regarding treatment recommendations. The recommendations are then communicated to the family and, when old enough, to the patient, by post, followed by an office consultation as required.

Ideally, as in all of healthcare, treatment should be based upon aetiology. Unfortunately, as with most of healthcare, treatment for VPD is only available for the consequences of an aetiology rather than the primary cause. While specific aetiologies can be associated with VPD, currently only one intervention for VPD specifically addresses the aetiology: intravelar veloplasty for congenital non-continuity of the velar muscular sling (sub-mucous cleft palate).[20] Most interventions for VPD create an non-physiologic state by placing an anatomic obstruction within the velo-pharyngeal port. This obstruction may be removable (speech prosthesis) or permanent (pharyngoplasty operations). The challenge for the velo-pharyngeal surgeon and the other VPD team members is to select which of the possible interventions will optimize outcome and minimize morbidity.

## THE VELO-PHARYNGEAL DYSFUNCTION MANAGEMENT ALGORITHM

**Figure 1:**
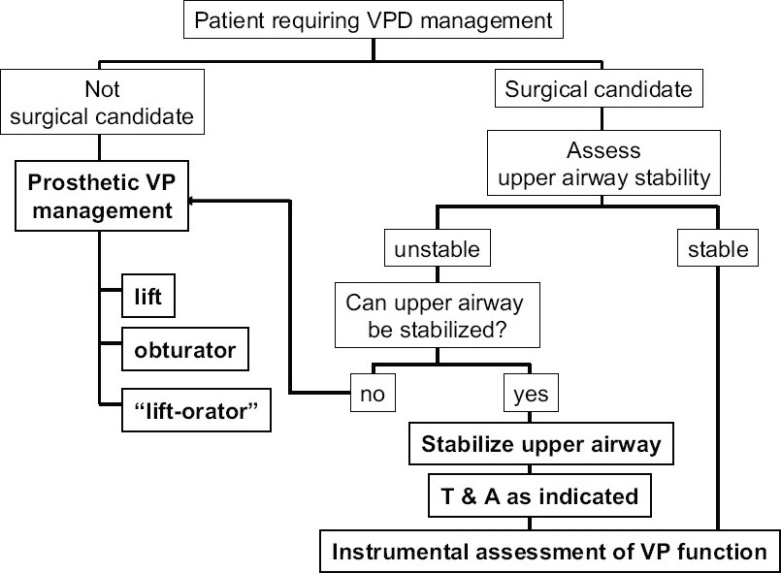
The initial decision tree for managing a patient with VPD is surgical candidacy and stability of the airway

**Figure 2:**
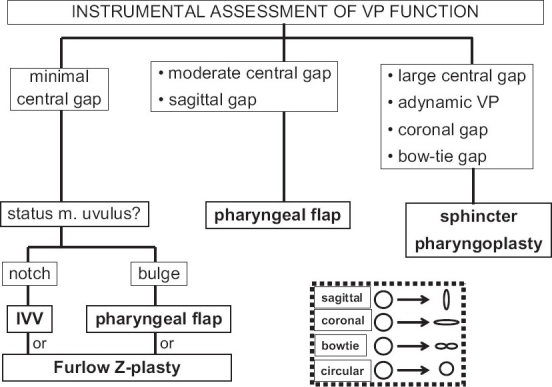
Differential surgical management of VPD is based upon the maximum closure pattern of the velopharyngeal port during specific speech tasks

Over the past 30 years, I have had the good fortune to work with two velo-pharyngeal management teams whose members were well-trained, experienced and with little turn-over. A VPD care algorithm formulated some 25 years ago[21] is still in use [Figures 1 and 2] followed by modifications with the introduction of new technology, unfamiliar operations, and lessons learned from other colleague via symposia and the literature. The basic principle of differential management for differential diagnosis has, however, remained constant.[22]

Once it has been determined that the patient has VPD due to an inability to achieve sufficient closure of the velo-pharyngeal port for normal nasal resonance and proper velo-pharyngeal related articulation, several sequential decisions are made to select the preferred means for VPD management.

1. The primary decision is whether or not the patient is a candidate for velo-pharyngeal surgery. Absolute contraindications to VPD surgery include: other congenital defects that unacceptably increase anaesthetic risk (such as uncorrected or uncorrectable cardiac or pulmonary disease), an unstable probably progressive neurological degenerative disorder, and parental opposition to surgery. Relative contraindications include a history of prior or concurrent obstructive sleep apnea, a small mandible, and an ectopic carotid artery (as can be seen in some 11q22 deletion patients).[23] Patients who require VPD management but are not deemed surgical candidates can be managed with a speech prosthesis.[24] The specific design of the prosthesis (palatal lift, speech bulb, or liftorator) depends upon the individual patient's velo-pharyngeal static and functional anatomy.[25,26] A palatal lift is prescribed for the patient with a long, unscarred velum; a speech bulb or obturator for the patient who has some degree of velo-pharyngeal sphincter movement but not sufficient for complete closure; a liftorator is a hybrid lift plus obturator for the patient who has sufficient velar length but insufficient lateral and posterior pharyngeal wall movement to achieve sufficient closure with a lift alone. A speech prosthesis is the preferred management for the neurologically unstable patient with VPD due to either a myoneurally deteriorating disease or a still resolving head injury.[27,28] A speech prosthesis also can be a diagnostic test for the benefit of surgical VPD management in the patient with a complex speech/language disorder.
2. If the patient is determined to be a candidate for surgical VPD management, the next decision would be the type of operation to be performed. Prior to discussion of the choice of operation, it is important to define the goals of intervention so that success and failure can be monitored. The simplistic goal of VPD management is to normalize speech. It is important to recognize that surgery per se cannot normalize speech; surgery can alter undesired movement of air and sound into the nose during speech. Elimination of the abnormal articulatory adaptations that an individual has made for this velo-pharyngeal escape in the attempt to produce intelligible speech requires behavioural modification therapy, i.e. speech therapy. Thus the individual with VPD without mal-articulations can have normal speech following velo-pharyngeal surgery without post-operative speech therapy. Most individuals with VPD, however, have some mal-articulations and therefore require post-operative speech therapy to achieve normal speech.[29] A more comprehensive goal of VPD surgery includes not only the elimination of the inappropriate nasal escape of air and sound but also maintenance of a patent nasal airway sufficient for both quiet nasal breathing with the lips closed and pharyngeal drainage of baseline nasal secretions.
3. Some surgeons only perform one operation for all patients with VPD. This is contrary to the basic healthcare principle of differential management based upon differential diagnosis. We, as others, therefore perform several different operations: individualizing intervention for each patient based upon consideration of a combination of the dynamics of the velo-pharyngeal port and the patient's other medical factors. For us, the two critical velo-pharyngeal function elements are the pattern of closure and the size of the residual gap. Of the four anatomic structures involved in velo-pharyngeal closure (velum, right and left lateral and posterior pharyngeal walls), the degree of movement of the two lateral walls is the prime determinant.
  - When lateral wall movement can close at least 50% of the distance from rest to anatomic sagittal midline, a narrow to medium width superiorly-based pharyngeal flap can be performed without concern of obstructing the nasal airway and inducing severe obstructive sleep apnoea.[30]
  - When lateral wall movement closure is less than 50% of the distance from position of rest to anatomic sagittal midline, we never perform a pharyngeal flap since in such cases a wide-obstructive flap is necessary to achieve normal speech with its attendant morbidity for the nasal airway and sleep apnoea.[31] Our procedure of choice for such cases is a sphincter pharyngoplasty. To express this in a slightly different way based upon velo-pharyngeal port closure patterns, we use a narrow to medium width superiorly-based pharyngeal flap for small to medium residual gaps (less than or equal to 50% of resting port cross-section area) that are sagittal or circular; we use a sphincter pharyngoplasty for large residual gaps (more than 50% of resting port cross-section area).
  - We had an unsatisfactory experience with autologous posterior pharyngeal wall augmentation for small gaps and no longer perform that operation.[32] We have no experience with alloplastic posterior pharyngeal wall augmentation having been discouraged by reports of extrusion or resorption.
  - Some authors report satisfactory management of small velo-pharyngeal residual gaps using a Furlow's Z-plasty[33,34] but we have no experience with this procedure.

## OUTCOME ASSESSMENT

There is no consensus regarding the method and timing of outcome assessment following VPD management. For decades, the protocol used by the velo- pharyngeal teams I have participated in has been a combination of perceptual and instrumental velo-pharyngeal function assessments at 3 and 12 months post-management. For many years, both perceptual and instrumental assessment (nasendoscopy and /or fluoroscopy) were performed at the three and 12 months evaluations. As with the pre-management assessments, these are audio/video recorded and archived for short-term and long-term comparison and study. Having learned the post-operative characteristics of both successful and unsuccessful types of VPD management, we now continue perceptual speech evaluations at 3 and 12 months post-operatively but restrict post-operative instrumental velo-pharyngeal functional visualization to those patients who have persistent symptomatic VPD. An annual audit collates the data from VPD managements of the prior 12 months for longitudinal intra-centre comparison as well as comparison to published outcome data from other centres[35–39]

Success is defined as a combination of the elimination of the symptomatic manifestations of the VPD (hypernasal resonance, nasal turbulence, and/or facial grimacing) and the maintenance of a sufficient nasal airway for quiet breathing with lips closed and secretion drainage.[40–42] While objective measurements of oral-nasal sound and airflow are useful research tools, how the individual sounds and looks during speech to lay-persons in the everyday environment is what matters for holistic outcome assessment.[43]

We have previously reported our experience with speech prostheses[25] pharyngeal flaps[44] sphincter pharyngoplasties[45] and autogenous posterior pharyngeal wall augmentations[32] for VPD management. No technique required transfusion or resulted in injury to the carotid arteries or death. Morbidity for both pharyngeal flap and sphincter pharyngoplasty consisted of persistent velopharyngeal dysfunction, impaired nasal secretion drainage, and obstructive sleep apnoea. Over the past 30 years, my utilization of each of the procedures for VPD management has altered as follows: speech prosthesis – decreased; pharyngeal flap- decreased; sphincter pharyngoplasty – increased; autogenous posterior pharyngeal wall augmentations – eliminated.

In separate reports of our series of 71 pharyngeal flaps[44] and 162 sphincter pharyngoplasties[23] between 1982 and 2000 with adequate preoperative and 3 and 12 month post-operative evaluations, resolution of hypernasality plus/minus nasal turbulence was: pharyngeal flap - initial is equal to 72%, secondary port tightening is equal to 92%; sphincter pharyngoplasty – initial is equal to 72%, secondary port tightening is equal to 85%, tertiary port tightening is equal to 100%. Significant hyponasality persisted at 12 months in 6% of pharyngeal flap patients and 10% of sphincter pharyngoplasty patients. Obstructive sleep apnoea was documented in seven per cent of pharyngeal flap patients and 14% of sphincter pharyngoplasty patients.

Between July 2003 and December 2008, my distribution of VPD management techniques was: speech prosthesis is equal to zero (two were referred for speech prosthesis but could not cooperate and later were managed surgically); pharyngeal flap is equal to three; sphincter pharyngoplasty is equal to 50. The resolution of hypernasality plus/minus nasal turbulence during this period was: pharyngeal flap – initial is equal to 100% (no post-operative obstructive sleep apneoa); sphincter pharyngoplasty – initial is equal to 96%, secondary port tightening is equal to 100%. Of the sphincter pharyngoplasties, 8/50 had post-operative obstructive sleep apnoea. Four of these had port stenosis that was resolved by surgical port enlargement in two. The remaining six had resolution of apnoea following uvulectomy with or without port debulking.

## THE CHALLENGE OF VPD MANAGEMENT IN DEVELOPING REGIONS OF THE WORLD

In at least three major areas, VPD management in developing regions differs from that in affluent and industrialized regions: 1. follow-up; 2. age at palatoplasty; 3. technology.

The successful performance of cleft lip and cleft palate repairs by both local and foreign surgeons in developing countries over the past several decades indicates the low morbidity of such operations in these environments. The peri-operative morbidity associated with surgical repair of cleft lip and cleft palate consists of bleeding, infection, dehiscence, fistula formation or tissue loss. While these may compromise the aesthetic and functional outcome of the operation, once healing is completed, the degree of morbidity does not progress. In contrast to the possible morbidity of cleft lip and/or palate surgery, too much narrowing of the velo- pharyngeal port can result in increasing symptomatology from nasal airway obstruction and/or obstructive sleep apnoea. When the patient who has undergone surgical velo- pharyngeal port diminution lives near the treating facility and returns for regular or problematic follow-up, these morbidities can be diagnosed and managed before causing more severe complications such as chronic sinusitis, poor school performance, diminished energy, enuresis, cor pulmonale and death. On the other hand, when the patient who has undergone surgical velo-pharyngeal port diminution lives far from the treating facility and does not return for follow-up, the complications of velo-pharyngeal port stenosis are often unrecognized and untreated causing further morbidity. The question that arises therefore is whether it is wise to offer VPD surgery in such situations. A search of literature did not seem to address this dilemma. Our practice in such environments is to leave the residual VP port(s) larger than we do in my own hospital for local patients with the surmise that it is better to have some residual VPD than to have obstruction of the nasal airway.

An additional difference between cleft-care in developed versus developing areas is the average older age of unrepaired patients. It is generally accepted that palatoplasty after 18 months of age in a normally developing individual is less likely to result in normal velo-pharyngeal function than when performed before 18 months of age. For this reason, some surgeons have advocated combining a primary pharyngeal flap with palatoplasty in the older, unrepaired, speaking individual. While we are unaware of any data to support or refute this practice, we subscribe to it when neither follow-up nor speech therapy is likely. In such cases, we inset a moderate-width, lined, superiorly-based pharyngeal flap into the velar nasal mucosa midline defect prior to intravelar veloplasty and oral mucosa repair; hard palate two-layer closure proceeds as with a plain palatoplasty.

In most developing areas there is a lack of technology for VP functional evaluation. As stated above, the diagnosis of VPD is made by auditory and visual perceptual assessment of speech. Differential diagnosis of the mechanism of the dysfunction can only be made with visualization of the VP port during specific speech tasks. Prior to the advent of dynamic fluoroscopic VP evaluation in the 1970's, surgical VP management was performed blindly in industrialized regions. This is what is often done today in developing areas. The author is unaware of any data to endorse or condemn blind VPD management in such environments. The author, however, advocates that while teaching VPD management operations to surgeons who lack the benefits of VP functional visualization in their diagnostic armamentarium, it should be with the expectation that they shall, at a later date, acquire the necessary diagnostic technology to aid them in differential management based upon differential diagnosis.

## CONCLUSION

**Figure 3:**
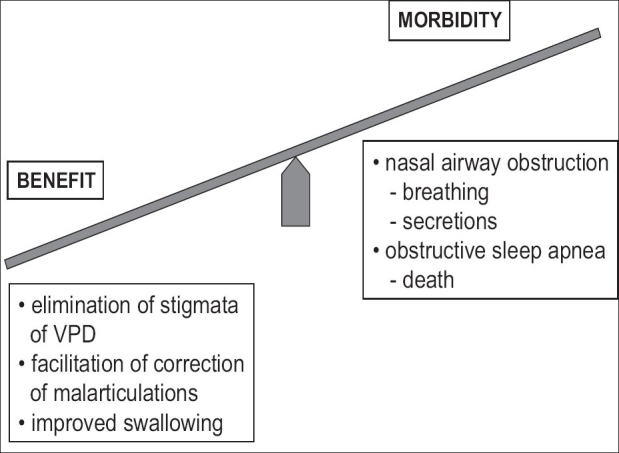
The benefits of VPD management should outweigh the morbidity of the intervention

Differential management of VPD based upon differential diagnosis of the dysfunction yields a high percentage of success with minimal morbidity. [Figures 3] When the patient has little probability of follow-up or access to sophisticated healthcare, more patent than usual post-operative VP port(s) with mild residual VPD may be preferable to a tight port(s) with probable nasal obstruction and sleep apnoea. The same approach is used when pre-operative VP functional visualization is unavailable. Although primary VP narrowing synchronous with palatoplasty is not advocated for younger children with unrepaired cleft palate, a primary pharyngeal flap with palatoplasty may be beneficial in the older unrepaired cleft palate individual.
